# Supplementary material for: High-quality metagenome assembly from nanopore reads with nanoMDBG
Source: Nat Commun. 2026 Mar 6;17:3556. doi: 10.1038/s41467-026-69760-y (PMC13087279; doi:10.1038/s41467-026-69760-y)
Supplement: Supplementary file 1 — Supplementary Information [file 41467_2026_69760_MOESM1_ESM.pdf]

Supplementary Information

Software	Version	Link
metaMDBG	1.1	https://github.com/GaetanBenoitDev/metaMDBG
metaMDBG asm –out-dir outputDir –in-ont reads –threads 16		
hifiasm-meta	0.3-r063.2	https://github.com/xfengnefx/hifiasm-meta
hifiasm_meta -t 16 -o outputDir/asm		
metaflye	2.9.3-b1797	https://github.com/fenderglass/Flye
flye –nano-hq reads –out-dir outputDir –threads 16 –plasmids –meta		
minimap2	2.28-r1209	https://github.com/lh3/minimap2
minimap2 -t 16 -x map-ont contigs reads		
samtools	1.2	https://github.com/samtools/
samtools sort -@ 16 -o align.bam		
semiBin2	2.1.0	https://github.com/BigDataBiology/SemiBin
SemiBin2 single_easy_bin –random-seed 42 –sequencing-type=long_read -p 16 -i contigFilename -b outputDir -o binDir –self-supervised –compression=none –tmpdir tmpDir		
CheckM2	1.0.1	https://github.com/chklovski/CheckM2
checkm2 predict –force –threads 16 -x fa -i binDir -o outputDir		
dRep	3.5.0	https://github.com/MrOlm/drep
dRep dereplicate outputDir -g binDir/*.fa -l 300000 –completeness 50 –contamination 100 -p 16 -sa 0.95 -1		
Infernal	1.1.5	https://github.com/EddyRivasLab/infernal
cmscan –cpu 16 –cut_ga –rfam –nohmmonly –fmt 2 –tblout outputFilename –clanin Rfam.clanin Rfam.cm magFilename		
Barrnap	0.9	https://github.com/tseemann/barrnap
barrnap –threads 16 –evalue 0.01 magFilename > outputFilename		
genomad	1.8.0	https://github.com/apcamargo/genomad
genomad end-to-end –splits 8 –conservative –cleanup contigFilename outputDir genomad_db –threads 16		
checkV	1.0.3	https://bitbucket.org/berkeleylab/checkv/src/master/
checkv end_to_end viralContigs.fasta resultDir -t 16		
wfmash	0.13	https://github.com/waveygang/wfmash
wfmash targetFilename queryFilename -t 16 > outputFilename		
herro	0.7.2	https://github.com/nanoporetech/dorado
dorado correct –model-path herro-v1/ -t 16 readFilename > outputFilename		
dechat	1.0.1	https://github.com/LuoGroup2023/DeChat
dechat -t 16 -i readFilename -o outputFilename		
ideel	1	https://github.com/mw55309/ideel
snakemake –cores 32 all		
anvi-script-find-misassemblies.py	8	https://anvio.org/help/main/programs/anvi-script-find-misassemblies/
anvi-script-find-misassemblies.py -b bamFilename -o clipFilename –just-do-it		
quast	5.2.0	https://github.com/ablab/quast
metaquast –unique-mapping –reuse-combined-alignments –threads 32 contigFilename -o outputDir -r referenceFilenames		

Supplementary Table 1: Software and command lines used. We give the software, version and download URL followed by precise command in following line.

Species	Assembler	Status	# mapped contigs	Completeness	Contiguity (auNGA)	# mismatches per 100 kbp	# indels per 100 kbp	# misassemblies
Akkermansia_muciniphila	nanoMDBG	single-contig	1	100.00%	2852392	6.14	4.14	0
2851480 bp	metaMDBG	-	4	99.93%	2698984	6.16	4.2	1
64x	metaflye	circular	1	100.00%	2851774	6.24	4	0
	hifiasm-meta	-	289	100.00%	1838393	28.19	17.1	2
Bacteroides_fragilis	nanoMDBG	single-contig	6	99.98%	5141629	9.91	3.99	1
5166942 bp	metaMDBG	single-contig	14	99.98%	3340546	22.14	20.54	3
334x	metaflye	circular	10	99.99%	3871443	7.03	4.87	5
	hifiasm-meta	-	1341	99.94%	1386814	51.74	25.77	67
Bifidobacterium_adolescentis	nanoMDBG	-	2	99.72%	1969903	7.42	2.68	2
2089645 bp	metaMDBG	-	7	99.86%	1969728	12.26	10.35	4
353x	metaflye	-	2	99.73%	1970679	7.37	2.49	1
	hifiasm-meta	-	123	99.17%	579749	23.77	8.97	3
Candida_albican	nanoMDBG	-	-	-	-	-	-	-
12575386 bp	metaMDBG	-	-	-	-	-	-	-
14x	metaflye	-	-	-	-	-	-	-
	hifiasm-meta	-	-	-	-	-	-	-
Clostridioides_difficile	nanoMDBG	-	5	99.97%	1546576	1.64	4.18	0
4209110 bp	metaMDBG	-	12	99.54%	1346305	8.85	11.67	7
43x	metaflye	-	7	99.86%	1233935	1.7	4.68	1
	hifiasm-meta	-	71	98.38%	1132376	5.26	3.38	1
Clostridium_perfringens	nanoMDBG	-	-	-	-	-	-	-
3436047 bp	metaMDBG	-	-	-	-	-	-	-
0x	metaflye	-	-	-	-	-	-	-
	hifiasm-meta	-	-	-	-	-	-	-
Enterococcus_faecalis	nanoMDBG	-	-	-	-	-	-	-
2845392 bp	metaMDBG	-	-	-	-	-	-	-
0x	metaflye	-	-	-	-	-	-	-
	hifiasm-meta	-	-	-	-	-	-	-
Escherichia_coli_B1109	nanoMDBG	-	26	53.83%	140642	123.34	5.73	4
4765434 bp	metaMDBG	-	28	73.34%	273759	120.31	19.5	4
72x	metaflye	-	50	46.48%	57418	187.9	16.5	2
	hifiasm-meta	-	134	77.52%	310983	69.24	14.37	11
Escherichia_coli_B3008	nanoMDBG	-	34	40.68%	55444	179.8	29.22	0
4739263 bp	metaMDBG	-	37	51.63%	55674	40.64	2.03	1
72x	metaflye	-	39	19.64%	23010	426.67	25.12	4
	hifiasm-meta	-	156	73.62%	550583	45.44	16.77	14
Escherichia_coli_B766	nanoMDBG	-	47	82.48%	98395	195.97	8.46	17
5062632 bp	metaMDBG	-	25	88.50%	195290	82.67	3.87	21
68x	metaflye	-	49	16.15%	6918	521.6	30.26	8
	hifiasm-meta	-	177	95.01%	284936	25.92	10.24	34
Escherichia_coli_JM109	nanoMDBG	-	23	48.95%	88533	93.45	5.44	9
4497410 bp	metaMDBG	-	19	32.28%	44092	50.47	7.7	1
76x	metaflye	-	33	30.87%	53294	96.09	12.05	3
	hifiasm-meta	-	123	62.93%	255589	54.04	20.28	5
Escherichia_coli_b2207	nanoMDBG	-	24	25.19%	22282	99.36	24.57	0
5111512 bp	metaMDBG	-	33	25.45%	34708	77.78	51.73	2
67x	metaflye	-	41	27.78%	27580	150.36	37	1
	hifiasm-meta	-	166	79.87%	262530	30.39	16.75	7
Faecalibacterium_prausnitzii	nanoMDBG	circular	4	100.00%	2918766	1.65	2.4	0
2913920 bp	metaMDBG	single-contig	14	100.00%	2910540	20.58	22.51	1
592x	metaflye	single-contig	4	100.00%	2909256	0.62	1.54	0
	hifiasm-meta	-	3187	100.00%	223588	35.11	19.05	109
Fusobacterium_nucleatum	nanoMDBG	circular	1	99.94%	2447330	1.1	2.57	2
2448186 bp	metaMDBG	-	18	99.80%	1564403	23.25	22.66	8
302x	metaflye	circular	1	99.98%	2446206	0.65	1.47	2
	hifiasm-meta	-	1860	99.32%	105632	31.6	12.44	68
Lactobacillus_fermentum	nanoMDBG	-	10	99.92%	1149517	18.83	2.83	1
1905333 bp	metaMDBG	-	21	98.98%	284268	21.13	5.6	1
388x	metaflye	-	15	99.90%	956893	19.02	2.1	0
	hifiasm-meta	-	339	90.99%	305354	46.16	21.73	34
Methanobrevibacter_smithii	nanoMDBG	-	56	87.53%	37015	31.67	31.85	7
1853160 bp	metaMDBG	-	63	77.56%	26384	23.54	26.23	7
6x	metaflye	-	53	82.11%	35134	66.72	73.62	2
	hifiasm-meta	-	40	67.32%	32086	124.69	116.13	5
Prevotella_corporis	nanoMDBG	-	4	99.45%	1776009	13.68	17.8	3
2947105 bp	metaMDBG	-	11	99.64%	2012326	33.66	39.52	2
250x	metaflye	-	5	99.58%	2187113	13.01	17.13	0
	hifiasm-meta	-	1569	99.19%	234120	40.24	32.99	39
Roseburia_hominis	nanoMDBG	circular	1	100.00%	3466856	1.85	3.38	0
3463215 bp	metaMDBG	circular	6	100.00%	3464293	2.74	4.18	0
498x	metaflye	single-contig	1	99.93%	3458616	1.24	2.46	0
	hifiasm-meta	-	1029	100.00%	1996456	25.05	11.66	1
Veillonella_rogosae	nanoMDBG	circular	1	100.00%	2160146	1.57	1.07	0
2158040 bp	metaMDBG	-	18	99.58%	1028631	26.24	22.19	0
799x	metaflye	circular	1	100.00%	2158046	1.62	0.93	0
	hifiasm-meta	-	1357	100.00%	614503	24.51	12.45	19
Salmonella_enterica	nanoMDBG	-	-	-	-	-	-	-
4759746 bp	metaMDBG	-	-	-	-	-	-	-
0x	metaflye	-	-	-	-	-	-	-
	hifiasm-meta	-	-	-	-	-	-	-
Saccharomyces_cerevisiae	nanoMDBG	-	-	-	-	-	-	-
12843354 bp	metaMDBG	-	-	-	-	-	-	-
14x	metaflye	-	-	-	-	-	-	-
	hifiasm-meta	-	-	-	-	-	-	-

Supplementary Table 2: Assembly results on Zymo mock community. We used MetaQUAST to compute metrics between the reference genomes and assemblies (see section “Quality assessment of assemblies on the Zymo mock community” for details). Completely absent rows (entirely -) mean that the reference is not present is the assembly. Status is “circular” if one circular contig covers at least 99% of the reference genome (single-contig if such contig is linear).

Dataset	Assembler	# bases	N50	>1Mb contigs	>1Mb near-complete contigs	>1Mb circular contigs	>1Mb near-complete circular contigs
ONT Human Gut (50 Gb)	nanoMDBG	546884976	348083	83	26	13	13
	metaMDBG	580396796	133752	67	15	11	11
	metaflye	512851952	159959	46	11	7	7
HiFi Human Gut (50 Gb)	metaMDBG	747399033	519816	136	38	23	22
	metaflye	766709414	342006	116	23	13	13
	hifiasm-meta	989961865	192957	154	38	27	25
ONT Zymo Fecal Reference (100 Gb)	nanoMDBG	1788080704	271195	250	71	35	34
	metaMDBG	1767712591	224269	236	64	38	36
	metaflye	1332968928	138924	88	29	18	18
ONT Zymo Fecal Reference (200 Gb)	nanoMDBG	2335383466	288676	349	83	41	40
	metaMDBG	2355332277	231062	332	77	46	43
	metaflye	1716979031	139796	120	44	24	24
HiFi Zymo Fecal Reference (100 Gb)	metaMDBG	1787378652	231603	228	57	40	38
	metaflye	1887778588	172674	155	39	19	19
	hifiasm-meta	2181882716	155263	229	46	33	29
HiFi Zymo Fecal Reference (200 Gb)	metaMDBG	2430598819	305001	346	88	47	46
	metaflye	2566737309	201472	256	61	36	36
ONT Soil (250 Gb)	nanoMDBG	21380430579	31407	673	41	25	20
	metaMDBG	18266511369	20628	246	5	4	4
	metaflye	16257389255	57584	192	1	0	0
HiFi Soil (250 Gb)	metaMDBG	24701035726	35868	875	73	39	34
	metaflye	17197167180	35170	91	3	1	1
	hifiasm-meta	22601685496	44433	284	17	17	12
ONT Soil (400 Gb)	nanoMDBG	31778238672	35656	1410	91	47	40
	metaMDBG	27742905062	21640	599	22	13	9
	metaflye	24820307022	62304	440	12	5	5

Supplementary Table 3: Quality assessment of assemblies (contigs). See manuscript section "Quality assessment of assemblies" for details on methods and software used.

Dataset	Assembler	# bases	N50	Near-complete MAGs	High-quality MAGs	Medium-quality MAGs	Near-Complete tRNA rRNA MAGs
ONT Human Gut (50 Gb)	nanoMDBG	546884976	348083	78	29	18	67
	metaMDBG	580396796	133752	55	32	27	46
	metaflye	512851952	159959	59	37	17	53
HiFi Human Gut (50 Gb)	metaMDBG	747399033	519816	114	27	25	102
	metaflye	766709414	342006	101	24	19	89
	hifiasm-meta	989961865	192957	75	40	35	66
ONT Zymo Fecal Reference (100 Gb)	nanoMDBG	1788080704	271195	190	94	67	162
	metaMDBG	1767712591	224269	167	83	73	145
	metaflye	1332968928	138924	135	84	46	118
ONT Zymo Fecal Reference (200 Gb)	nanoMDBG	2335383466	288676	255	109	96	223
	metaMDBG	2355332277	231062	225	104	93	198
	metaflye	1716979031	139796	167	112	65	149
HiFi Zymo Fecal Reference (100 Gb)	metaMDBG	1787378652	231603	204	106	73	178
	metaflye	1887778588	172674	149	76	66	129
	hifiasm-meta	2181882716	155263	120	88	112	109
HiFi Zymo Fecal Reference (200Gb)	metaMDBG	2430598819	305001	294	112	92	259
	metaflye	2566737309	201472	228	116	86	205
ONT Soil (250 Gb)	nanoMDBG	21380430579	31407	166	149	158	157
	metaMDBG	18266511369	20628	23	50	80	21
	metaflye	16257389255	57584	74	175	188	67
HiFi Soil (250 Gb)	metaMDBG	24701035726	35868	165	158	172	158
	metaflye	17197167180	35170	23	27	52	20
	hifiasm-meta	22601685496	44433	27	35	77	26
ONT Soil (400 Gb)	nanoMDBG	31778238672	35656	260	221	235	246
	metaMDBG	27742905062	21640	59	82	140	52
	metaflye	24820307022	62304	116	267	235	109

Supplementary Table 4: Quality assessment of assemblies (MAGs). See manuscript section "Quality assessment of assemblies" for details on methods and software used.

Dataset	Assembler	Virus	Circular Virus	High-quality Circular Virus	Plasmids	Circular Plasmids
ONT Human Gut (50 Gb)	nanoMDBG	595	24	23	100	36
	metaMDBG	660	22	20	135	30
	metaflye	591	27	23	193	27
HiFi Human Gut (50 Gb)	metaMDBG	720	31	29	111	42
	metaflye	628	25	21	116	19
	hifiasm-meta	1171	32	28	448	24
ONT Zymo Fecal Reference (200 Gb)	nanoMDBG	2735	61	45	411	90
	metaMDBG	2862	52	40	447	79
	metaflye	2540	121	74	468	66
HiFi Zymo Fecal Reference (200 Gb)	metaMDBG	2821	86	68	348	83
	metaflye	3102	93	56	682	62
ONT Soil (250 Gb)	nanoMDBG	8711	418	243	1789	13
	metaMDBG	7243	178	107	1703	13
	metaflye	3947	280	148	887	12
HiFi Soil (250 Gb)	metaMDBG	9178	572	315	2203	20
	metaflye	2989	139	70	636	10
	hifiasm-meta	3385	445	257	956	18
ONT Soil (400 Gb)	nanoMDBG	13364	714	424	2837	26
	metaMDBG	11712	349	215	2590	21
	metaflye	6634	477	261	1605	17

Supplementary Table 5: Number of plasmids and virus identified by genomad in each assembly. We used checkV to assess the quality of viral circular contigs (see manuscript section "Quality assessment of assemblies" for details.

Dataset	Assembler	Run time (h)	Peak memory (GB)
ONT Human Gut (50 Gb)	nanoMDBG	5	17
	metaMDBG	6	11
	metaflye	8	114
HiFi Human Gut (50 Gb)	metaMDBG	3	11
	metaflye	4	78
	hifiasm-meta	35	222
ONT Zymo Fecal Reference (200 Gb)	nanoMDBG	46	30
	metaMDBG	39	29
	metaflye	34	395
HiFi Zymo Fecal Reference (200 Gb)	metaMDBG	15	13
	metaflye	28	379
ONT Soil (250 Gb)	nanoMDBG	73	73
	metaMDBG	48	68
	metaflye	355	485
HiFi Soil (250 Gb)	metaMDBG	64	68
	metaflye	260	514
	hifiasm-meta	67	580
ONT Soil (400 Gb)	nanoMDBG	149	125
	metaMDBG	106	110
	metaflye	680	752

Supplementary Table 6: Computational performance of the assemblers.

Rank	Assembler	Name	Number of MAGs
genus	nanoMDBG	QUBU01	1
genus	nanoMDBG	Chryseolinea	3
genus	nanoMDBG	genus_ukwn_18	1
genus	nanoMDBG	JADGRB01	3
genus	nanoMDBG	Halioglobus	1
genus	nanoMDBG	PMG-095	2
genus	nanoMDBG	genus_ukwn_1	1
genus	nanoMDBG	JACCZR01	1
genus	nanoMDBG	genus_ukwn_36	2
genus	nanoMDBG	CADEED01	3
genus	nanoMDBG	SZUA-320	1
genus	nanoMDBG	genus_ukwn_52	1
genus	nanoMDBG	RPQJ01	3
genus	nanoMDBG	JAJKJG01	1
genus	nanoMDBG	DP-20	1
genus	nanoMDBG	genus_ukwn_35	1
genus	nanoMDBG	genus_ukwn_38	1
genus	nanoMDBG	genus_ukwn_56	1
genus	nanoMDBG	genus_ukwn_4	1
genus	nanoMDBG	genus_ukwn_5	1
genus	nanoMDBG	JAAYBF01	1
genus	nanoMDBG	GMQP-bins7	3
genus	nanoMDBG	genus_ukwn_40	1
genus	nanoMDBG	genus_ukwn_41	1
genus	nanoMDBG	genus_ukwn_53	1
genus	nanoMDBG	genus_ukwn_42	2
genus	nanoMDBG	JACVRW01	2
genus	nanoMDBG	FEN-1219	1
genus	nanoMDBG	JJ008	1
genus	nanoMDBG	Gemmatimonas	1
genus	nanoMDBG	Dongia_A	1
genus	nanoMDBG	JAICEW01	1
genus	nanoMDBG	Thiobacillus	1
genus	nanoMDBG	PALSA-612	2
genus	nanoMDBG	genus_ukwn_30	1
genus	nanoMDBG	Lacipirellula	1
genus	nanoMDBG	genus_ukwn_28	1
genus	nanoMDBG	Humibacillus	1
genus	nanoMDBG	Terrimicrobium	2
genus	nanoMDBG	Promineifilum	2
genus	nanoMDBG	genus_ukwn_17	1
genus	nanoMDBG	Nitrospira_A	1
genus	nanoMDBG	OLB17	2
genus	nanoMDBG	JACDBZ01	1
genus	nanoMDBG	genus_ukwn_15	1
genus	nanoMDBG	JACCXG01	1
genus	nanoMDBG	genus_ukwn_9	1
genus	nanoMDBG	JALZAA01	1
genus	nanoMDBG	CADEFD01	2
genus	nanoMDBG	JABDGM01	1
genus	nanoMDBG	JAGPDF01	1
genus	nanoMDBG	JAAYXG01	1
genus	nanoMDBG	JAOUNA01	1
genus	nanoMDBG	genus_ukwn_13	1
genus	nanoMDBG	SXOA01	1
genus	nanoMDBG	SPCO01	2
genus	nanoMDBG	CADEFX01	1

genus	nanoMDBG	Luteitalea	2
genus	nanoMDBG	genus_ukwn_3	1
genus	nanoMDBG	genus_ukwn_2	1
genus	nanoMDBG	genus_ukwn_47	1
genus	nanoMDBG	CANJBX01	1
genus	nanoMDBG	genus_ukwn_7	1
genus	nanoMDBG	CALGFH01	1
genus	nanoMDBG	JAHFTY01	1
genus	nanoMDBG	JACMLA01	1
genus	nanoMDBG	Lapillicoccus	2
genus	nanoMDBG	PLA5	1
genus	nanoMDBG	Schlegelella_A	1
genus	nanoMDBG	Microbacterium	2
genus	nanoMDBG	genus_ukwn_33	1
genus	nanoMDBG	genus_ukwn_32	1
genus	nanoMDBG	Nitrospira_C	1
genus	nanoMDBG	UBA4720	1
genus	nanoMDBG	Intrasporangium	1
genus	nanoMDBG	AG11	1
genus	nanoMDBG	Rubrivivax_A	1
genus	nanoMDBG	UBA8639	1
genus	nanoMDBG	genus_ukwn_26	1
genus	nanoMDBG	JADGNW01	1
genus	nanoMDBG	JAICNR01	1
genus	nanoMDBG	genus_ukwn_31	1
genus	nanoMDBG	genus_ukwn_10	1
genus	nanoMDBG	genus_ukwn_25	1
genus	metaMDBG	Rubrobacter_D	1
genus	metaMDBG	JAIBJP01	1
genus	metaMDBG	CALGFI01	1
genus	metaMDBG	genus_ukwn_24	1
genus	metaflye	NS-11	1
genus	metaflye	PALSA-1355	1
genus	metaflye	SXYR01	1
genus	metaflye	JAFAEM01	1
genus	metaflye	BPGH01	1
genus	metaflye	AR5	1
genus	metaflye	genus_ukwn_44	1
genus	metaflye	genus_ukwn_48	1
genus	metaflye	JAQGOX01	1
genus	metaflye	genus_ukwn_46	1
genus	metaflye	JACVRX01	1
genus	metaflye	genus_ukwn_57	1
genus	metaflye	Serratia_A	1
genus	metaflye	genus_ukwn_11	1
genus	metaflye	genus_ukwn_27	1
genus	metaflye	JADIYM01	1
genus	metaflye	genus_ukwn_39	1
genus	metaflye	Mesorhizobium_F	1
genus	metaflye	CALKUS01	1
genus	metaflye	genus_ukwn_19	1
genus	metaflye	genus_ukwn_23	1
genus	metaflye	Pseudorhodoplanes	1
family	nanoMDBG	GCA-2729495	1
family	nanoMDBG	Cyclobacteriaceae	3
family	nanoMDBG	Fen-1088	4
family	nanoMDBG	Halieaceae	1
family	nanoMDBG	Polyangiaceae	2

family	nanoMDBG	Thermoleophilaceae	4
family	nanoMDBG	JAEUHO01	1
family	nanoMDBG	Beijerinckiaceae	1
family	nanoMDBG	UBA9968	1
family	nanoMDBG	RSA9	1
family	nanoMDBG	UTPRO1	1
family	nanoMDBG	PALSA-555	1
family	nanoMDBG	JAEUJM01	1
family	nanoMDBG	family_ukwn_7	1
family	nanoMDBG	Rariloculaceae	2
family	nanoMDBG	Dongiaceae	1
family	nanoMDBG	Thiobacillaceae	1
family	nanoMDBG	Solirubrobacteraceae	1
family	nanoMDBG	Lacipirellulaceae	2
family	nanoMDBG	Dermatophilaceae	4
family	nanoMDBG	Terrimicrobiaceae	2
family	nanoMDBG	Promineifilaceae	2
family	nanoMDBG	QHBO01	1
family	nanoMDBG	JACDBD01	1
family	nanoMDBG	SMWR01	1
family	nanoMDBG	JAKEFK01	1
family	nanoMDBG	Pirellulaceae	1
family	nanoMDBG	Hyphomicrobiaceae	1
family	nanoMDBG	Vicinamibacteraceae	2
family	nanoMDBG	UBA6930	1
family	nanoMDBG	family_ukwn_0	1
family	nanoMDBG	Gemmataceae	1
family	nanoMDBG	CALGFH01	1
family	nanoMDBG	20CM-2-55-15	2
family	nanoMDBG	Bryobacteraceae	1
family	nanoMDBG	PLA5	1
family	nanoMDBG	Microbacteriaceae	2
family	nanoMDBG	UBA8639	1
family	nanoMDBG	family_ukwn_2	1
family	nanoMDBG	UBA7656	1
family	nanoMDBG	family_ukwn_4	1
family	metaMDBG	Rubrobacteraceae	1
family	metaflye	NS-4	1
family	metaflye	PALSA-610	1
family	metaflye	JAHEKZ01	1
family	metaflye	Planctomycetaceae	1
family	metaflye	JAQGOX01	1
family	metaflye	Pan216	1
family	metaflye	Jiangellaceae	1
family	metaflye	B-1AR	1
family	metaflye	Enterobacteriaceae	1
family	metaflye	JADIYM01	1
family	metaflye	Rhizobiaceae	1
family	metaflye	Ahniellaceae	1
order	nanoMDBG	GCA-2729495	1
order	nanoMDBG	Cytophagales	3
order	nanoMDBG	Fen-1088	4
order	nanoMDBG	Pseudomonadales	1
order	nanoMDBG	Polyangiales	2
order	nanoMDBG	PLA2	1
order	nanoMDBG	UBA9968	1
order	nanoMDBG	Longimicrobiales	1
order	nanoMDBG	UTPRO1	1

order	nanoMDBG	RBG-16-71-46	1
order	nanoMDBG	Rariloculales	2
order	nanoMDBG	Dongiales	1
order	nanoMDBG	Actinomycetales	6
order	nanoMDBG	Promineifilales	2
order	nanoMDBG	QHBO01	1
order	nanoMDBG	UBA9160	2
order	nanoMDBG	JAKEFK01	1
order	nanoMDBG	UBA796	1
order	nanoMDBG	Gemmatales	1
order	nanoMDBG	CALGFH01	1
order	nanoMDBG	20CM-2-55-15	2
order	nanoMDBG	Bryobacterales	1
order	nanoMDBG	Polarisedimenticolales	1
order	metaMDBG	Rubrobacterales	1
order	metaflye	JAHEKZ01	1
order	metaflye	Planctomycetales	1
order	metaflye	Limisphaerales	1
order	metaflye	Pan216	1
order	metaflye	Jiangellales	1
order	metaflye	SJA-28	1
order	metaflye	Enterobacterales	1
order	metaflye	Xanthomonadales	1
class	nanoMDBG	Polyangia	6
class	nanoMDBG	PLA2	1
class	nanoMDBG	UBA9160	2
class	nanoMDBG	HRBIN11	1
class	nanoMDBG	UBA796	1
class	nanoMDBG	CALGFH01	1
class	nanoMDBG	Terriglobia	3
class	nanoMDBG	Polarisedimenticolia	1
class	metaMDBG	Rubrobacteria	1
class	metaflye	Ignavibacteria	1
phylum	nanoMDBG	Myxococcota_A	2

Supplementary Table 7: List of taxa recovered from the ONT-Soil dataset found by only one of the assemblers.

Dataset	Assembler	Clipping events >10x coverage	Clipping events >10x coverage per 100Mbp	Regions larger than 1000 bp with zero coverage	Regions larger than 1000 bp with zero coverage per 100Mbp
ONT Human Gut	nanoMDBG	13	2.38 (50 Gb)	29	5.30
	metaMDBG	20	3.45	131	22.57
	metaflye	83	16.18	38	7.41
HiFi Human Gut	metaMDBG	16	2.14 (50 Gb)	169	22.61
	metaflye	16	2.09	163	21.26
	hifiasm-meta	4	0.40	23	2.32
ONT Zymo Fecal Reference (100 Gb)	nanoMDBG	91	5.09	380	21.25
	metaMDBG	70	3.96	748	42.31
	metaflye	418	31.36	319	23.93
HiFi Zymo Fecal Reference (100 Gb)	metaMDBG	54	3.02	371	20.76
	metaflye	72	3.81	409	21.67
	hifiasm-meta	9	0.41	30	1.37
ONT Soil (250 Gb)	nanoMDBG	642	3.00	6199	28.99
	metaMDBG	1339	7.33	5730	31.37
	metaflye	2101	12.92	7157	44.02
HiFi Soil (250 Gb)	metaMDBG	673	2.72	12727	51.52
	metaflye	1489	8.66	20700	120.37
	hifiasm-meta	33	0.15	807	3.57

Supplementary Table 8: Evaluation of clipping events and zero coverage regions in metagenomics assemblies. Results are shown for all assemblers and data sets.

Dataset	Assembler	IDEEL predicted proteins	IDEEL full-length protein fraction	>1Mb contaminated contigs	>1Mb contaminated contigs fraction
ONT Human Gut (50 Gb)	nanoMDBG	323340	0.887	1	0.012
	metaMDBG	355260	0.866	4	0.06
	metaflye	324647	0.862	1	0.022
HiFi Human Gut (50 Gb)	metaMDBG	422426	0.896	2	0.015
	metaflye	436824	0.892	0	0
	hifiasm-meta	608873	0.873	6	0.039
ONT Zymo Fecal Reference (100 Gb)	nanoMDBG	976757	0.874	5	0.02
	metaMDBG	1046790	0.878	6	0.025
	metaflye	835219	0.832	1	0.011
HiFi Zymo Fecal Reference (100 Gb)	metaMDBG	930553	0.889	1	0.004
	metaflye	1024149	0.885	1	0.006
	hifiasm-meta	1267894	0.874	6	0.026
ONT Soil (250 Gb)	nanoMDBG	5884382	0.702	7	0.01
	metaMDBG	6475751	0.676	7	0.028
	metaflye	7075382	0.569	0	0
HiFi Soil (250 Gb)	metaMDBG	5619483	0.765	6	0.007
	metaflye	5558032	0.704	0	0
	hifiasm-meta	5512900	0.686	1	0.004

Supplementary Table 9: Evaluation of IDEEL predicted proteins and contaminated contigs across metagenomics assemblies. Results are shown for all assemblers and data sets.

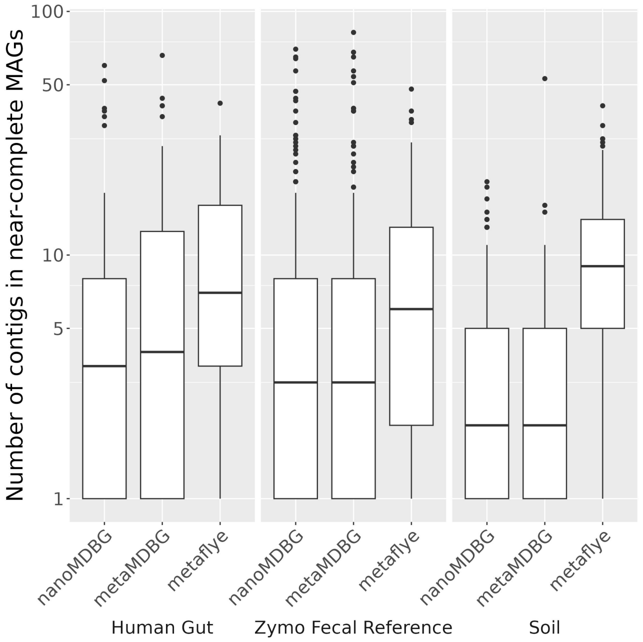

Supplementary Figure 1: Number of contigs in near-complete MAGs across the three ONT samples. The boxplot elements are the median (horizontal bar), 25th and 75th percentiles (box limits Q1 and Q3), Q1-1.5*IQR and Q3+1.5*IQR (whiskers, IQR=Q3-Q1) and outliers. Summary statistics (n, min, median, mean, max): Human gut - nanoMDBG (78, 1, 3.5, 7.6, 60); metaMDBG (55, 1, 4, 10, 66); metaFlye (59, 1, 7, 10.6, 42) : Zymo Fecal Reference - nanoMDBG (254, 1, 3, 2.3, 70); metaMDBG (225, 1, 3, 2.3, 82); metaFlye (167, 1, 6, 8.8, 48) : Soil - nanoMDBG (260, 1, 2, 1.3, 20); metaMDBG (59, 1, 2, 2.3, 53); metaFlye (116, 1, 9, 10.5, 41).

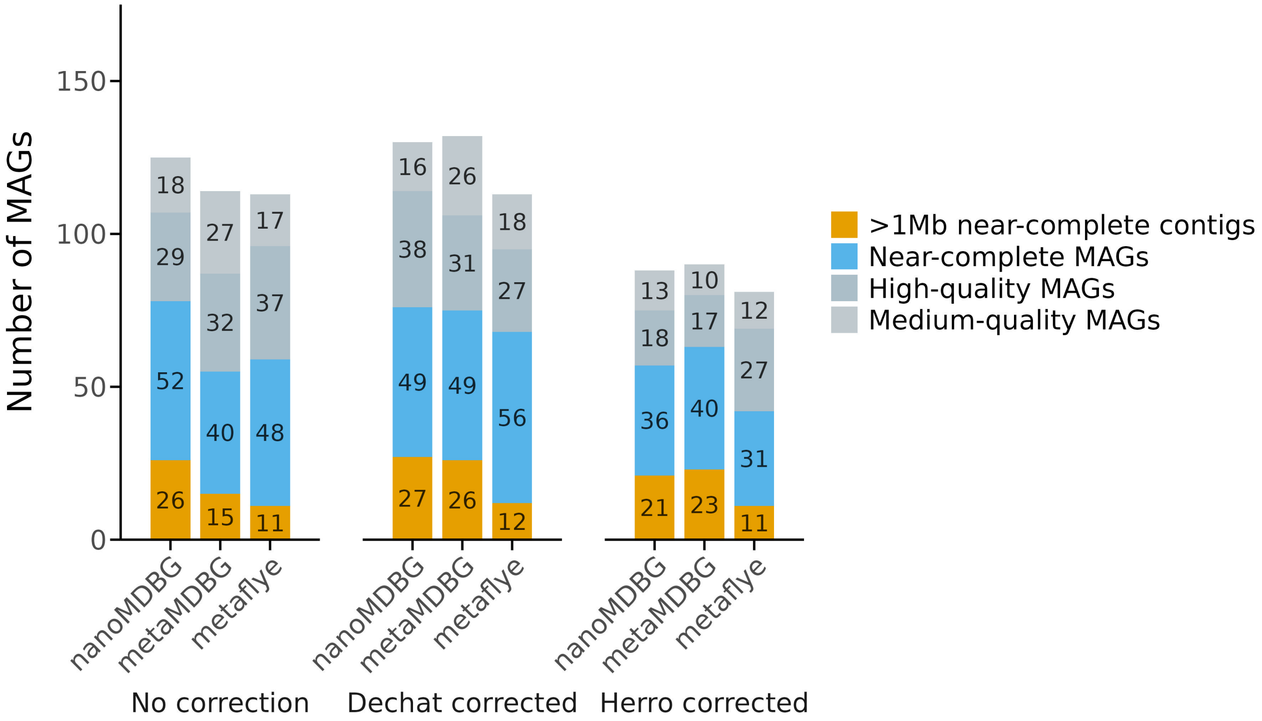

Supplementary Figure 2: Assembly results on ONT Human gut sample using preliminary base-level correction methods. We ran DeChat and HERRO correction tools on the 50 Gb Human gut sample, followed by assembly with nanoMDBG, metaMDBG and metaFlye. "No correction" corresponds to the assembly results on the raw read sets.

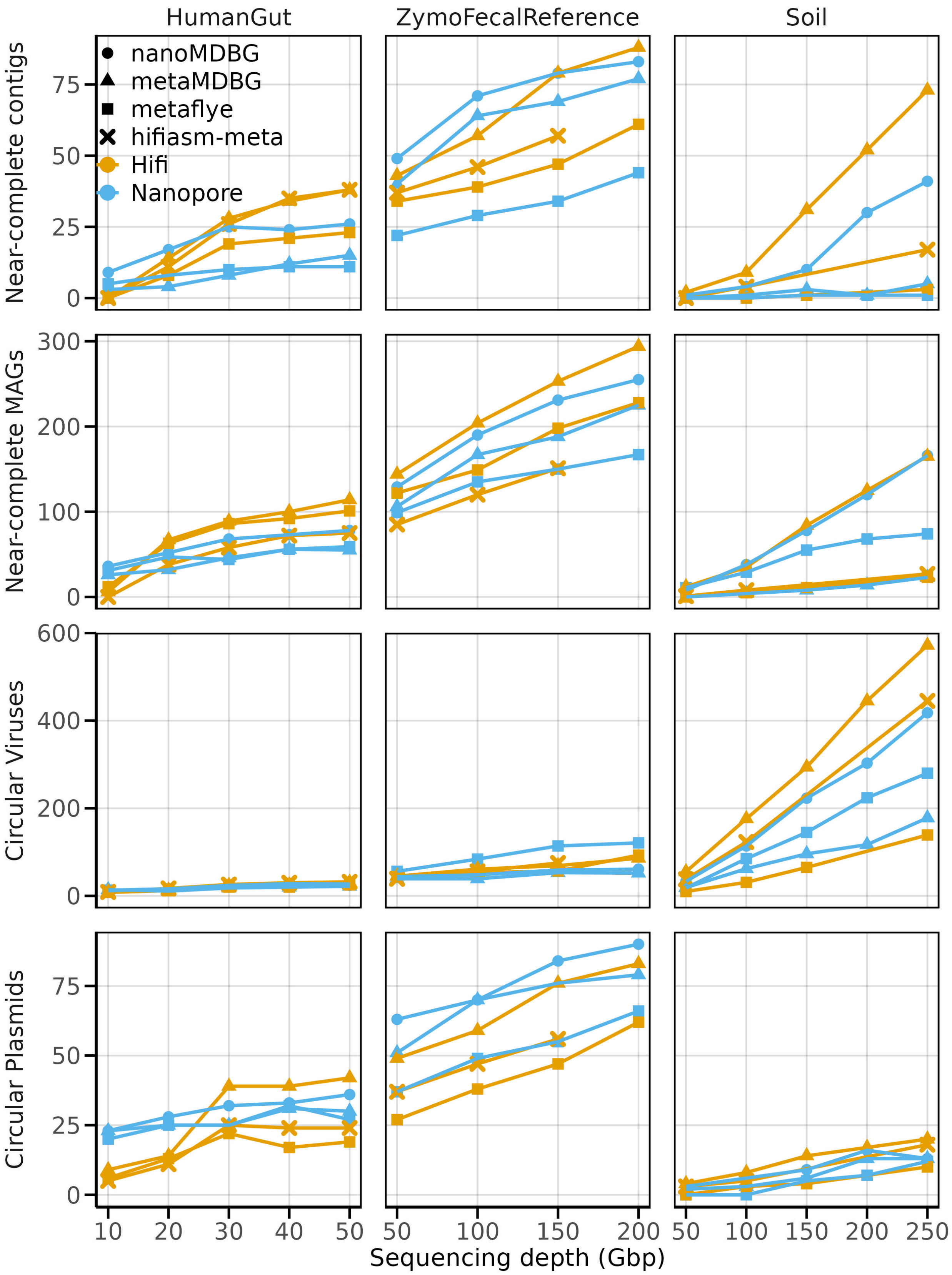

Supplementary Figure 3: Summary of assembly results for every assembler for both ONT and HiFi data generated from the same samples. Results are shown for three samples, Human Gut, the Zymo Fecal Reference and Soil. The x-axis gives sequencing depth in Gbp generated by randomly subsampling reads and the y-axis number of contigs that were near-complete prokaryotic genomes (near-complete contigs), near-complete prokaryotic MAGs following binning, and circular viruses and plasmids respectively.
